# Supplementary figures and images for: Species-specific enhancement of enterohemorrhagic E. coli pathogenesis mediated by microbiome metabolites
Source: Microbiome. 2019 Mar 20;7:43. doi: 10.1186/s40168-019-0650-5 (PMC6425591; doi:10.1186/s40168-019-0650-5)

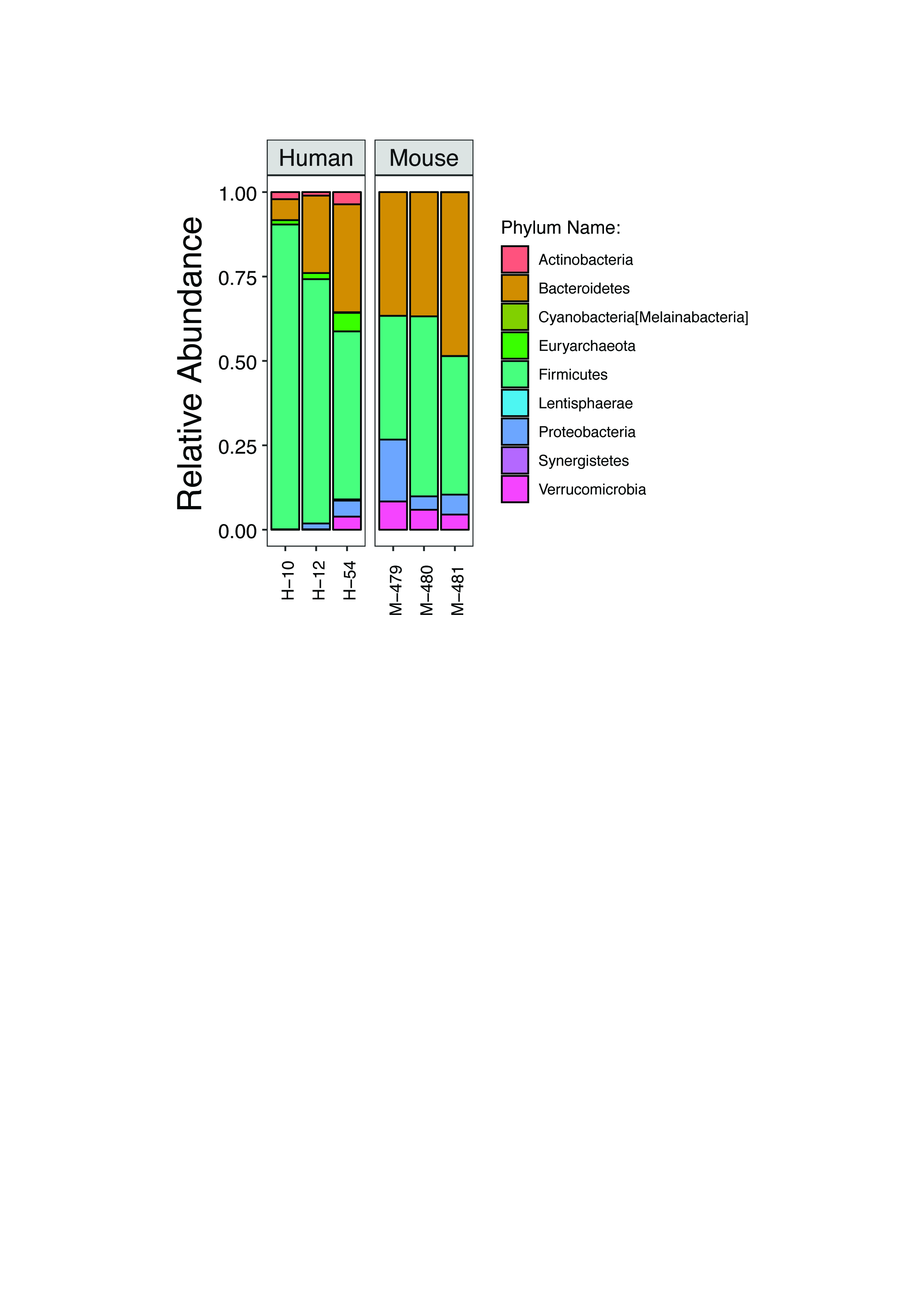

Supplement: Supplementary file 1 — Figure S1. Analysis of relative abundance of PolyFermS commensal bacteria phyla. Relative abundance of phyla measured in the last stage of human and murine microbial fermentation in PolyFermS. (TIF 2104 kb) [file 40168_2019_650_MOESM1_ESM.tif]

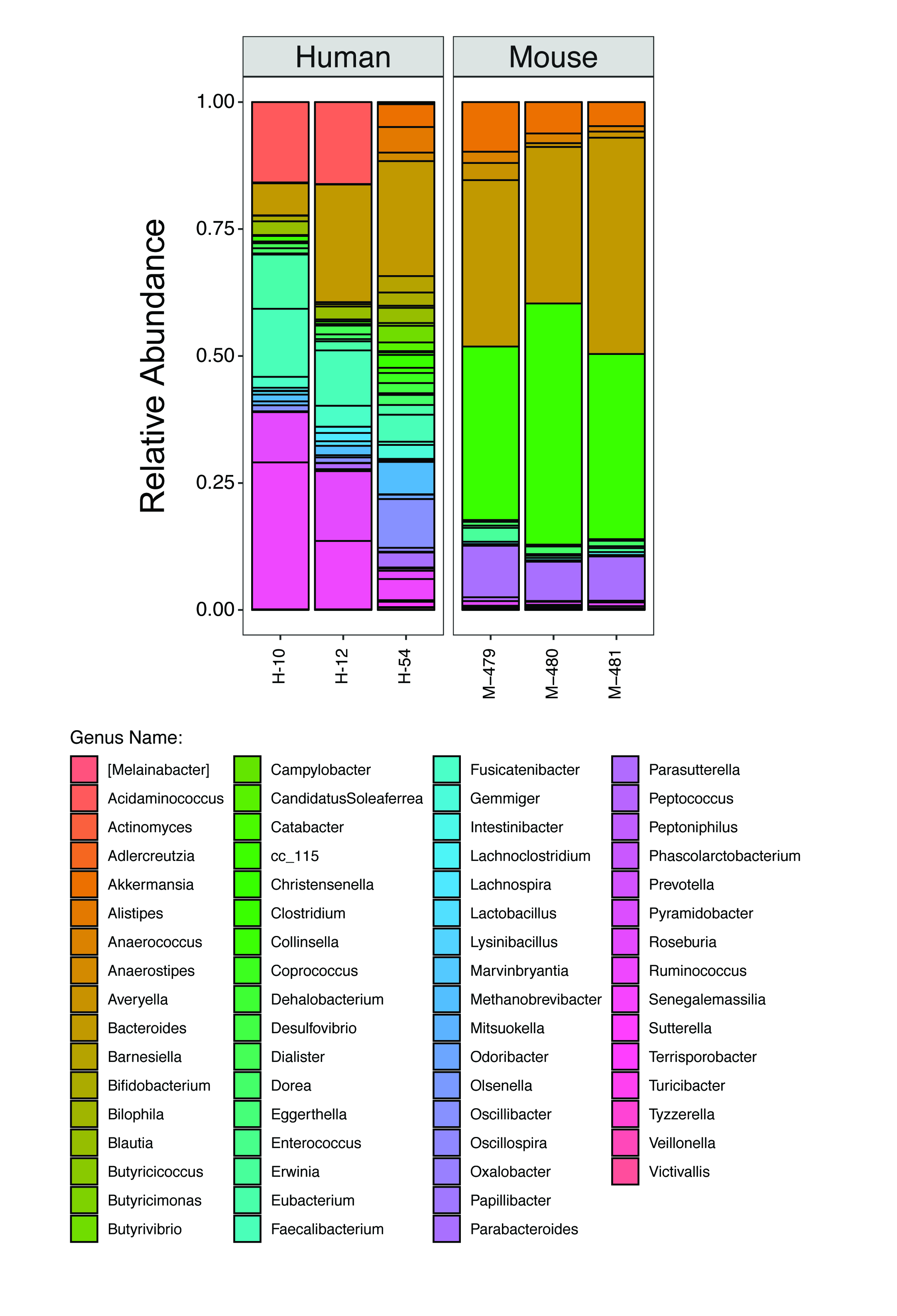

Supplement: Supplementary file 2 — Figure S2. Analysis of relative abundance of PolyFermS commensal bacteria genera. Relative abundance of genera measured in the last stage of human and murine microbial fermentation in PolyFermS. (TIF 3460 kb) [file 40168_2019_650_MOESM2_ESM.tif]

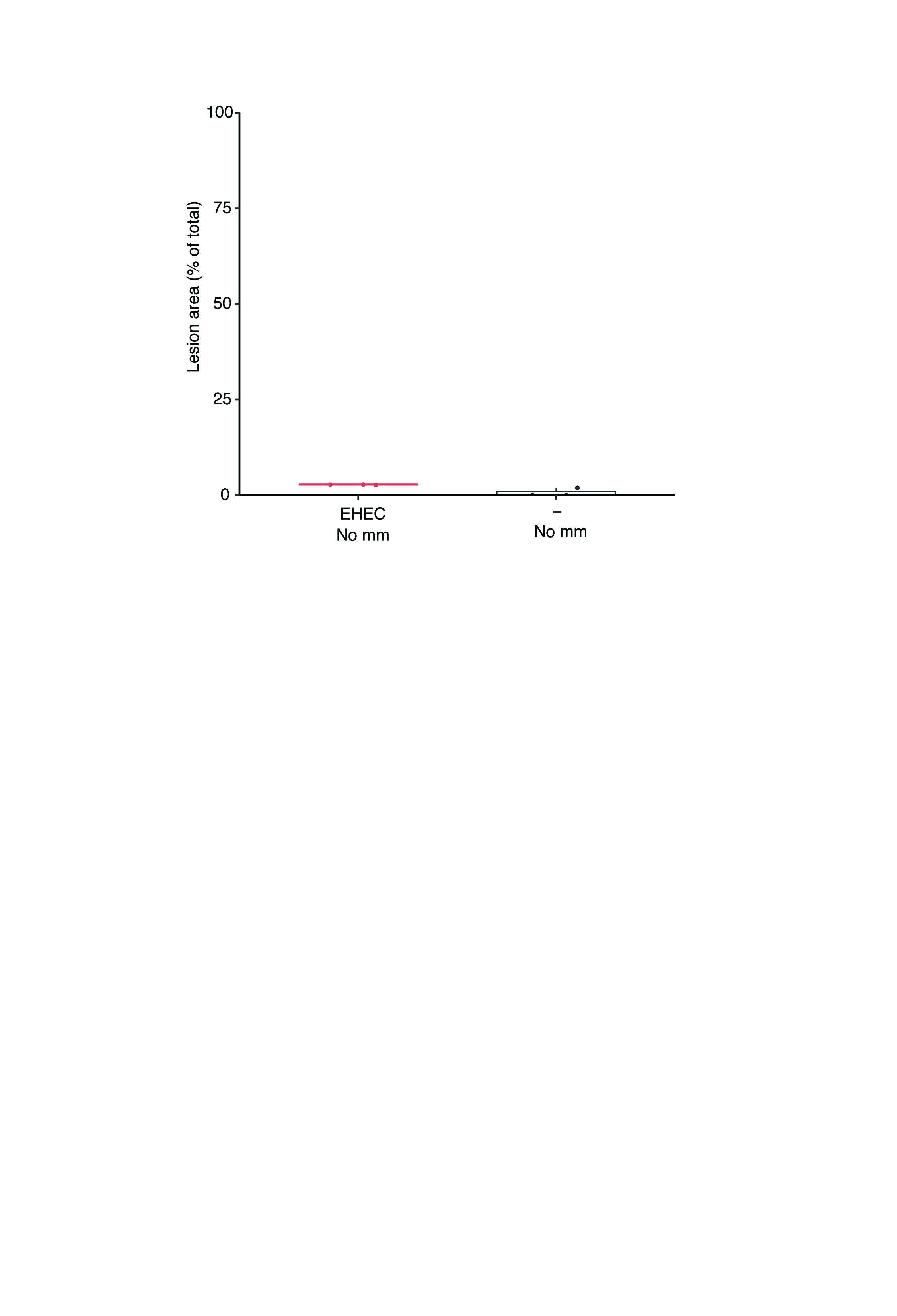

Supplement: Supplementary file 3 — Figure S3. EHEC infection of Colon Chips in the absence of microbial metabolites. Analysis of EHEC-induced epithelial injury on-chip. (TIF 1389 kb) [file 40168_2019_650_MOESM3_ESM.tif]

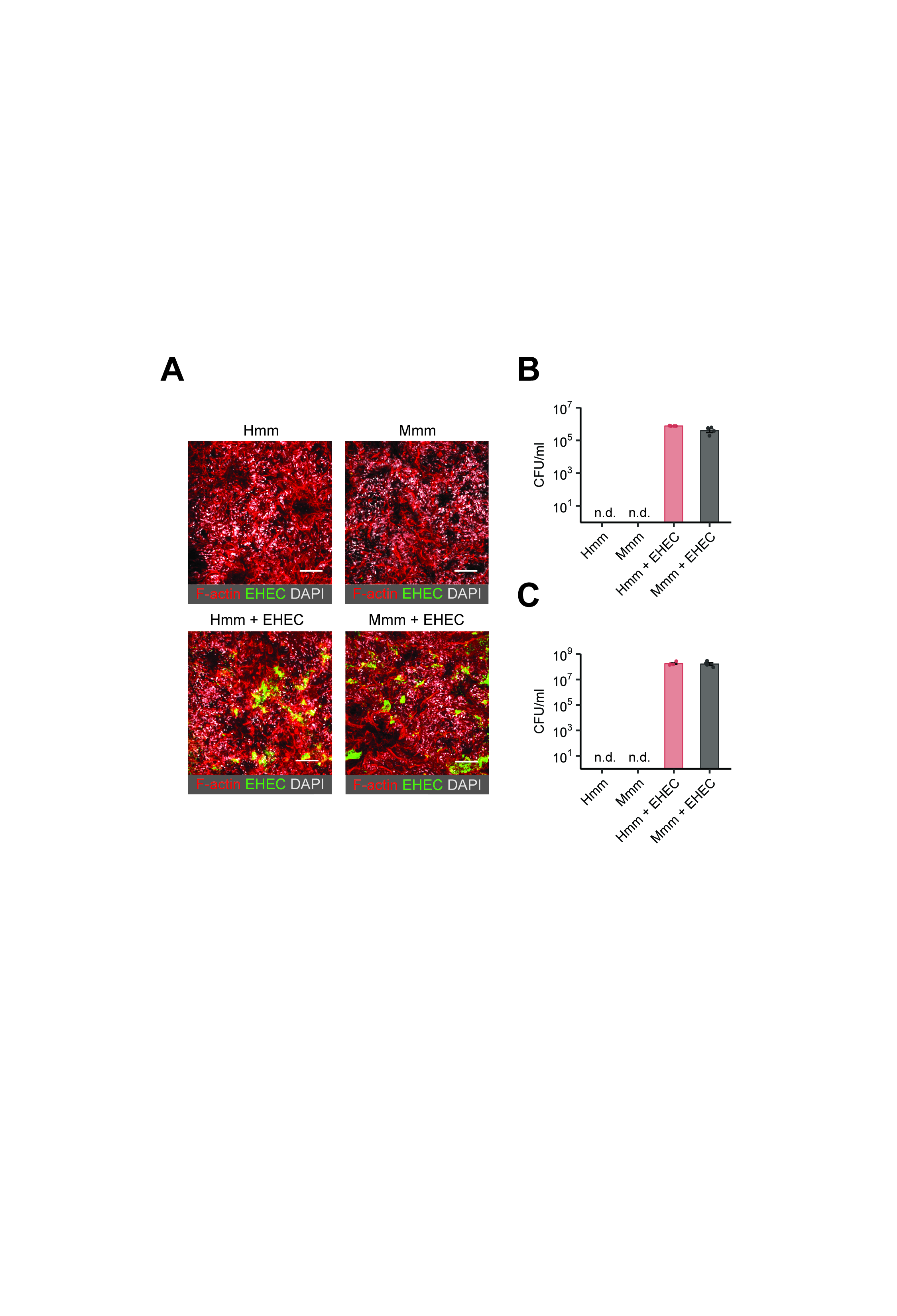

Supplement: Supplementary file 4 — Figure S4. Species-specific injury effects are not due to changes in EHEC colonization. (A-C) EHEC colonization of the human Colon Chip. (A) Representative fluorescence images showing the epithelial layer of infected and control Colon Chips in the presence or absence of Hmm or Mmm, with or without EHEC present (red: F-actin, green: GFP-EHEC, white: nuclei; bar, 100 μm). (B) Quantification of EHEC bacteria adherent to the intestinal epithelium. (C) Quantification of non-adherent EHEC quantification floating in the culture medium (TIF 5673 kb) [file 40168_2019_650_MOESM4_ESM.tif]

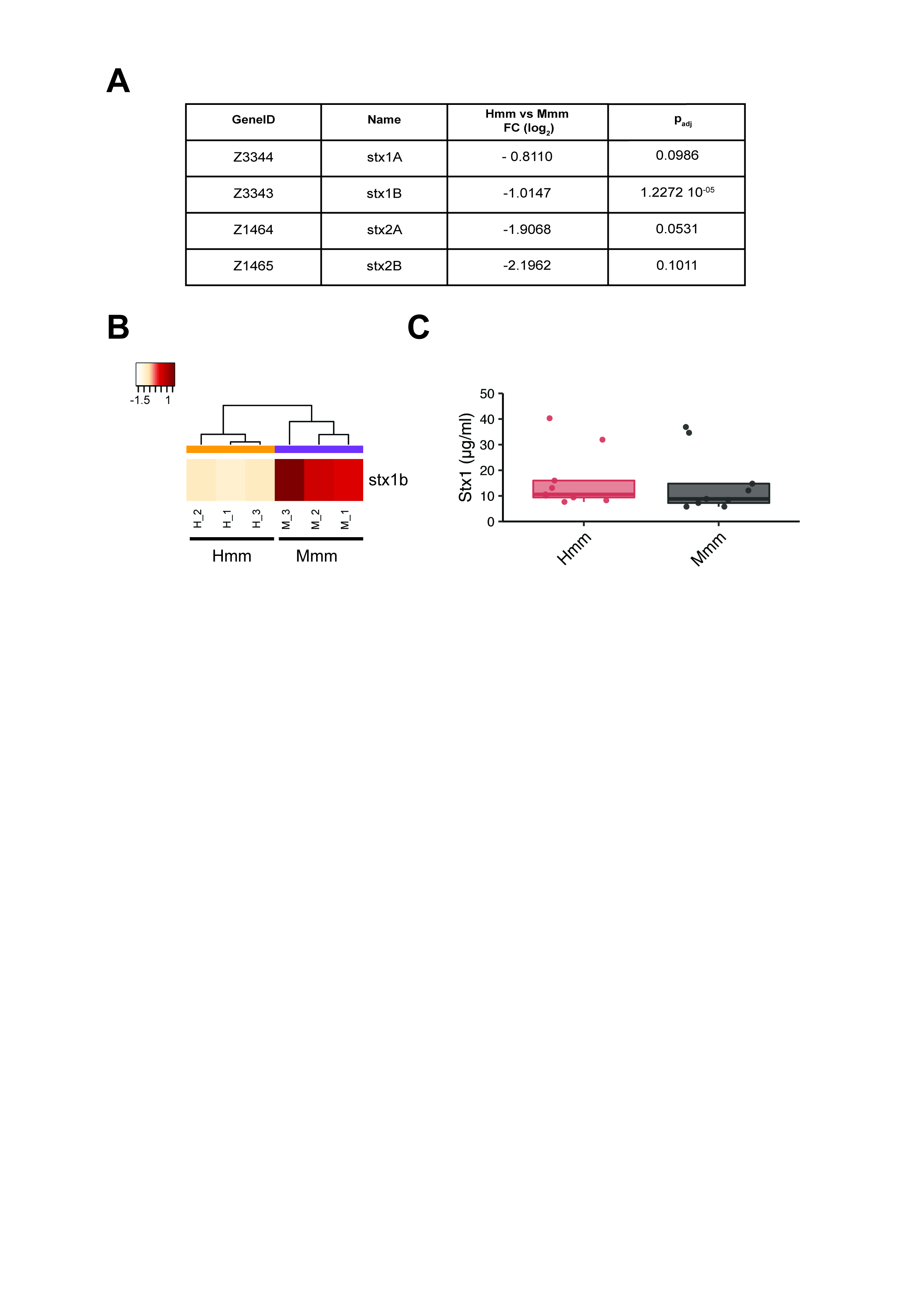

Supplement: Supplementary file 6 — Figure S5. Species-specific injury effects are not due to Shiga toxin. (A) Table with transcriptomics comparison of EHEC shiga toxin genes from Hmm and Mmm Colon Chips (FC: fold change). (B) Heatmap of the differentially expressed gene stx1b. (C) Quantification of shiga toxin one released in the vascular channel of Colon Chips. (TIF 1494 kb) [file 40168_2019_650_MOESM6_ESM.tif]

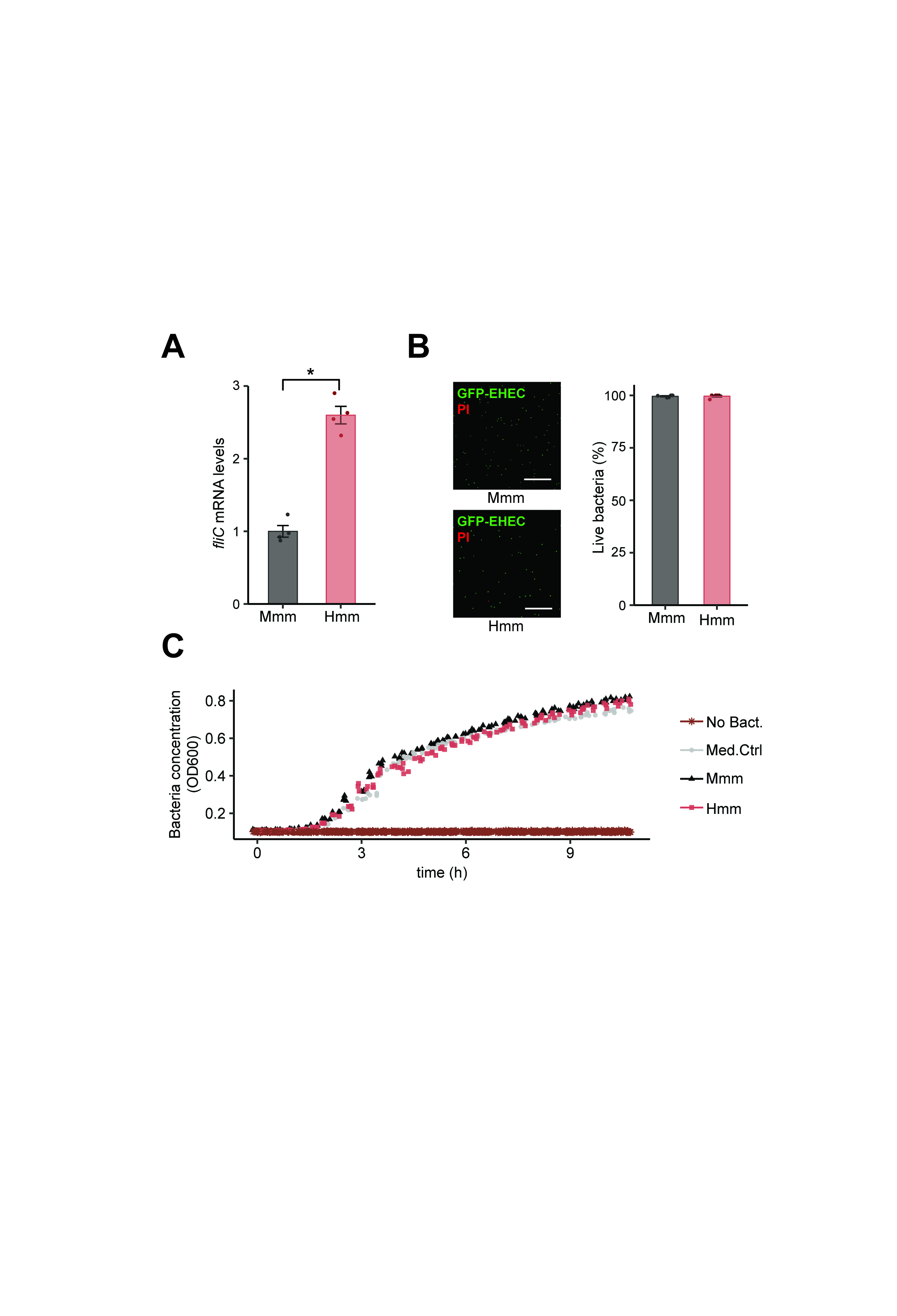

Supplement: Supplementary file 7 — Figure S6. FliC gene transcript is upregulated by Hmm, the species-specific motility effect is not due to changes bacteria viability, and the fliC-luciferase increase in the presence of Hmm is not due to altered bacterial growth. (A) FliC mRNA levels in EHEC cultured with Hmm or Mmm (shown as linearized, normalized fold change). (B) Fluorescence microscopic image of GFP-EHEC (green) and quantification of EHEC viability by staining with propidium iodide (red; bar, 100 μm). (C) Bacterial concentration determined as optical density measured at 600 nm (OD600) of EHEC fliC-luciferase in the presence of Mmm or Mmm. (TIF 1939 kb) [file 40168_2019_650_MOESM7_ESM.tif]

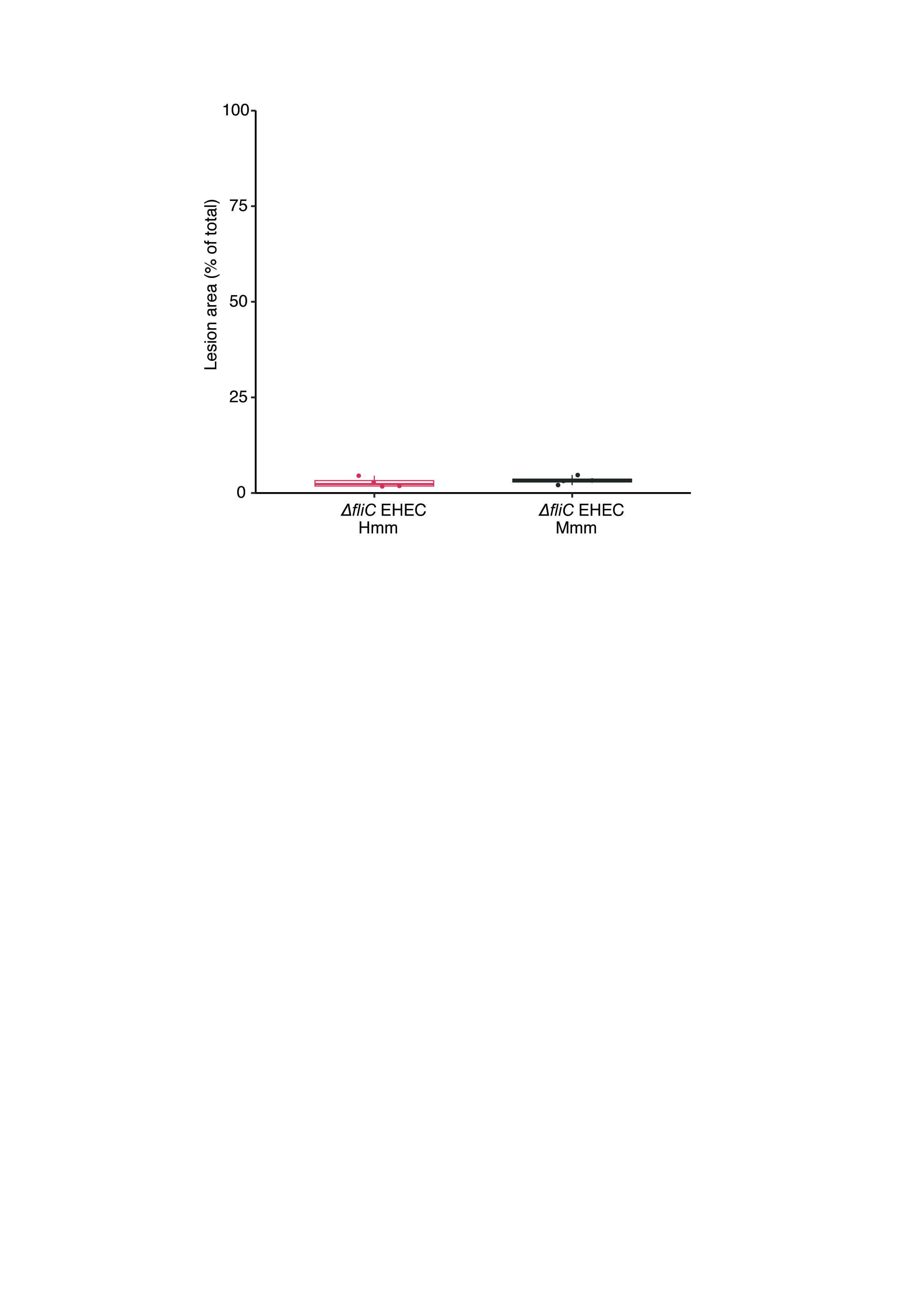

Supplement: Supplementary file 8 — Figure S7. ΔfliC EHEC does not produce differential epithelial lesions in the Hmm and Mmm groups. Analysis of ΔfliC EHEC induced epithelial injury on-chip. (TIF 1400 kb) [file 40168_2019_650_MOESM8_ESM.tif]

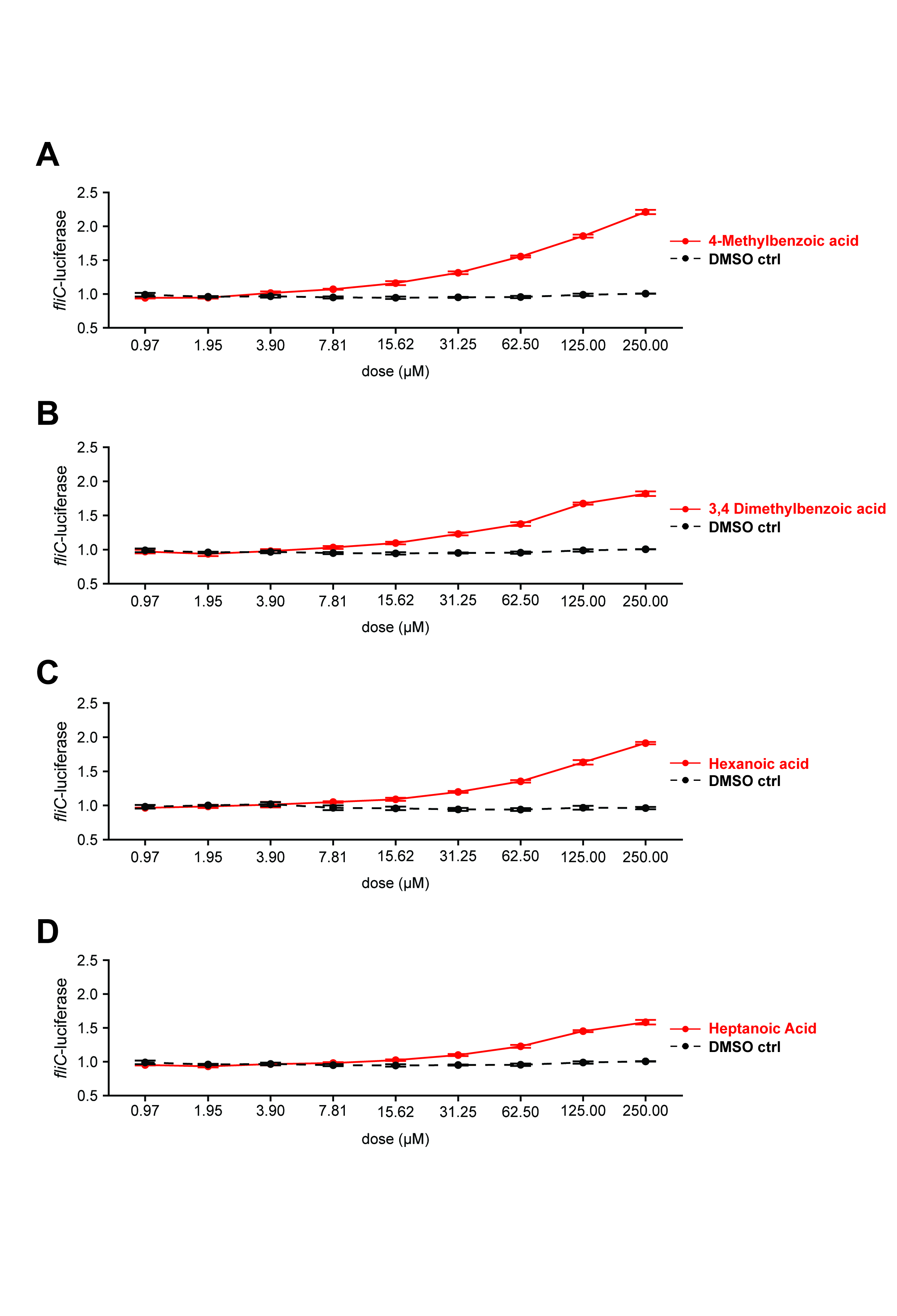

Supplement: Supplementary file 10 — Figure S8. Each of the 4 identified metabolites increases fliC expression in a dose-dependent manner. FliC-luciferase levels (determined by quantifying the AUC and normalizing for the DMSO control) of 4-methylbenzoic acid, 3,4 dimethylbenzoic acid, hexanoic acid, and heptanoic acid metabolites measured at indicated concentrations. (TIF 1914 kb) [file 40168_2019_650_MOESM10_ESM.tif]

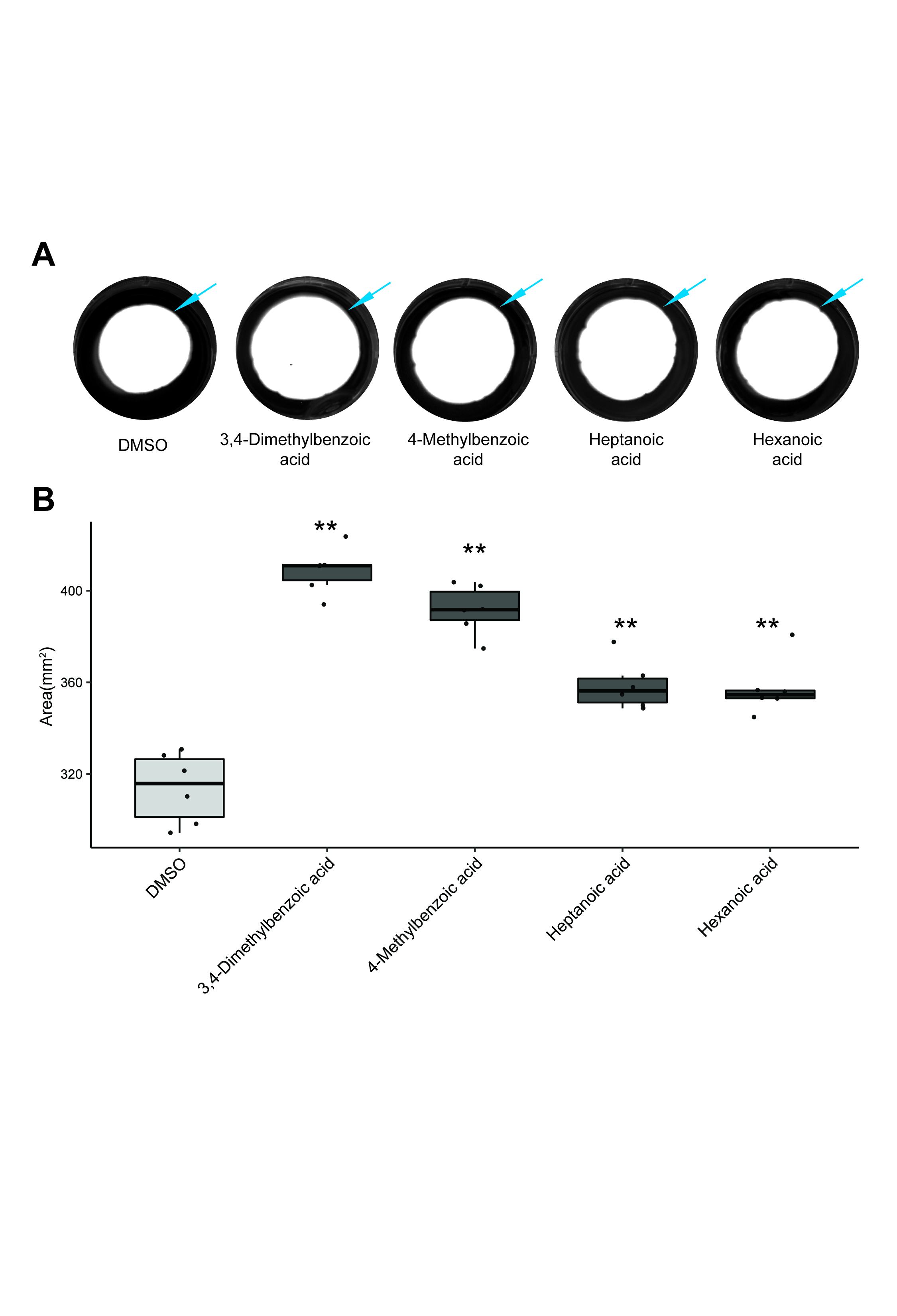

Supplement: Supplementary file 11 — Figure S9. The 4 identified active metabolites increase EHEC motility in a plate-based swimming assay. (A, B). Effects of 3,4-dimethylbenzoic acid, 4-methylbenzoic acid, hexanoic acid, and heptanoic acid (all at 200 μM) individually on EHEC-GFP swimming motility. (A) Photographic image of the plate containing EHEC-GFP bacteria (white) cultured with each of the 4 metabolites (black: plate background; blue arrows indicate the edge of the area occupied by bacteria). (B) Quantification of the area occupied by EHEC-GFP in A. **p < 0.01. (TIF 2040 kb) [file 40168_2019_650_MOESM11_ESM.tif]

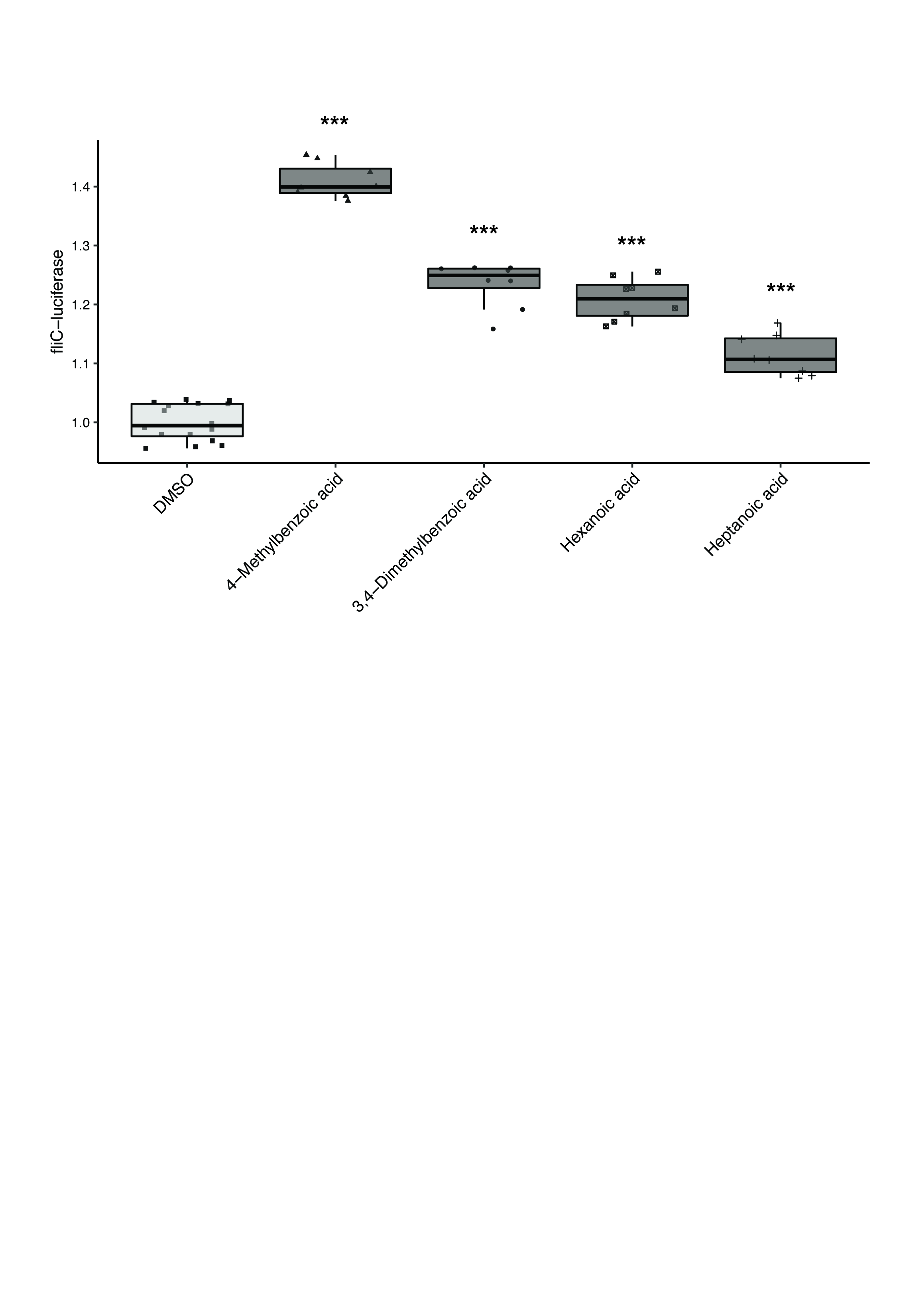

Supplement: Supplementary file 12 — Figure S10. Each of the 4 identified metabolites increases fliC expression in EHEC serotype O91:H21. FliC-luciferase levels (determined by quantifying the AUC and normalizing for the DMSO control) of 4-methylbenzoic acid, 3,4 dimethylbenzoic acid, hexanoic acid, and heptanoic acid metabolites at a concentration of 200 μM. ***p < 0.001 (TIF 1525 kb) [file 40168_2019_650_MOESM12_ESM.tif]

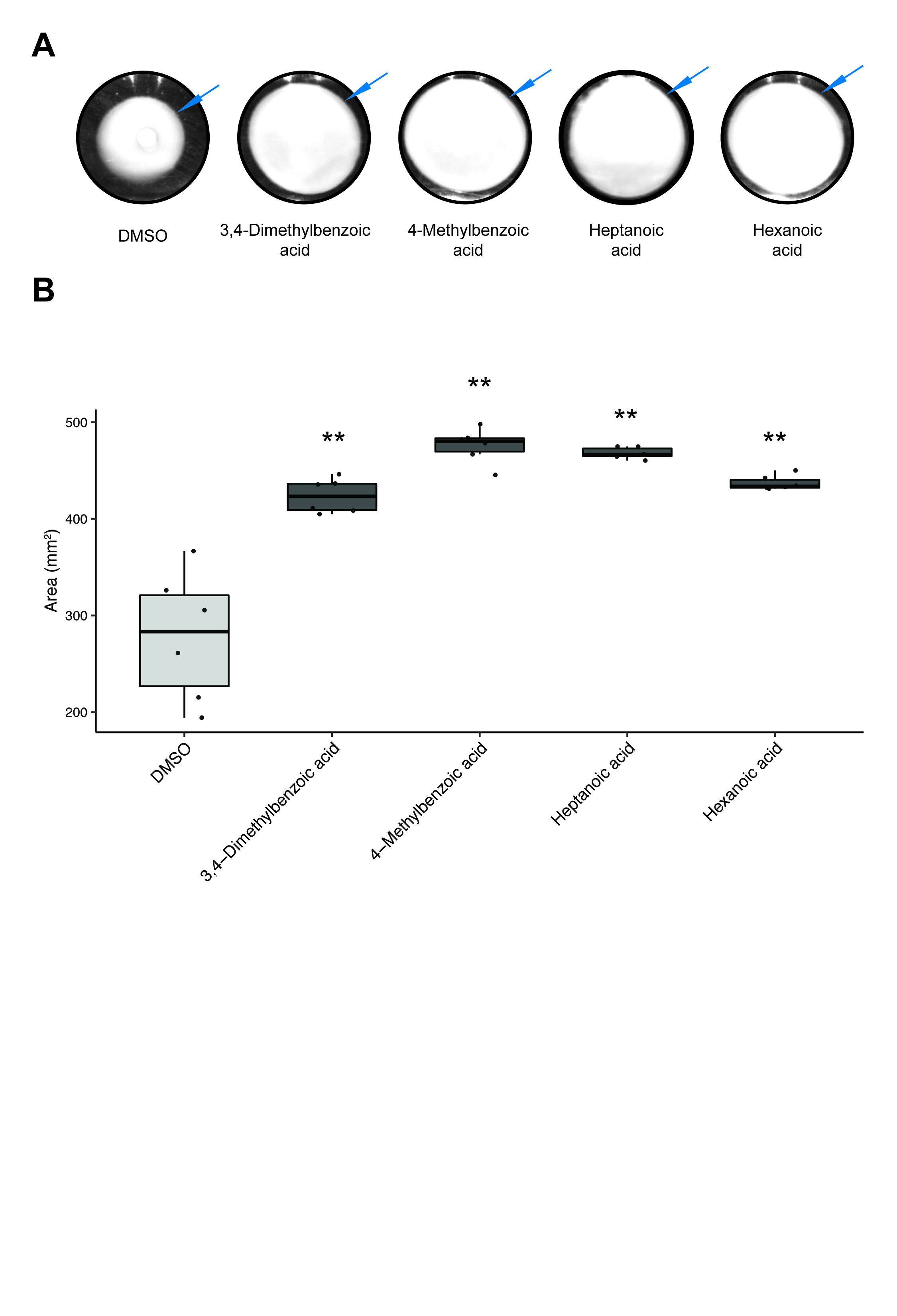

Supplement: Supplementary file 13 — Figure S11. The 4 identified active metabolites increase motility in a plate-based swimming assay in EHEC serotype O91:H21. (A, B) Effects of 3,4-dimethylbenzoic acid, 4-methylbenzoic acid, hexanoic acid, and heptanoic acid (all at 200 μM) individually on EHEC (serotype O91:H21) swimming motility. (A) Bright field photographic image of the plate containing EHEC bacteria (white) cultured with each of the 4 metabolites (black: plate background; blue arrows indicate the edge of the area occupied by bacteria). (B) Quantification of the area occupied by EHEC in A. **p < 0.01. (TIF 1998 kb) [file 40168_2019_650_MOESM13_ESM.tif]

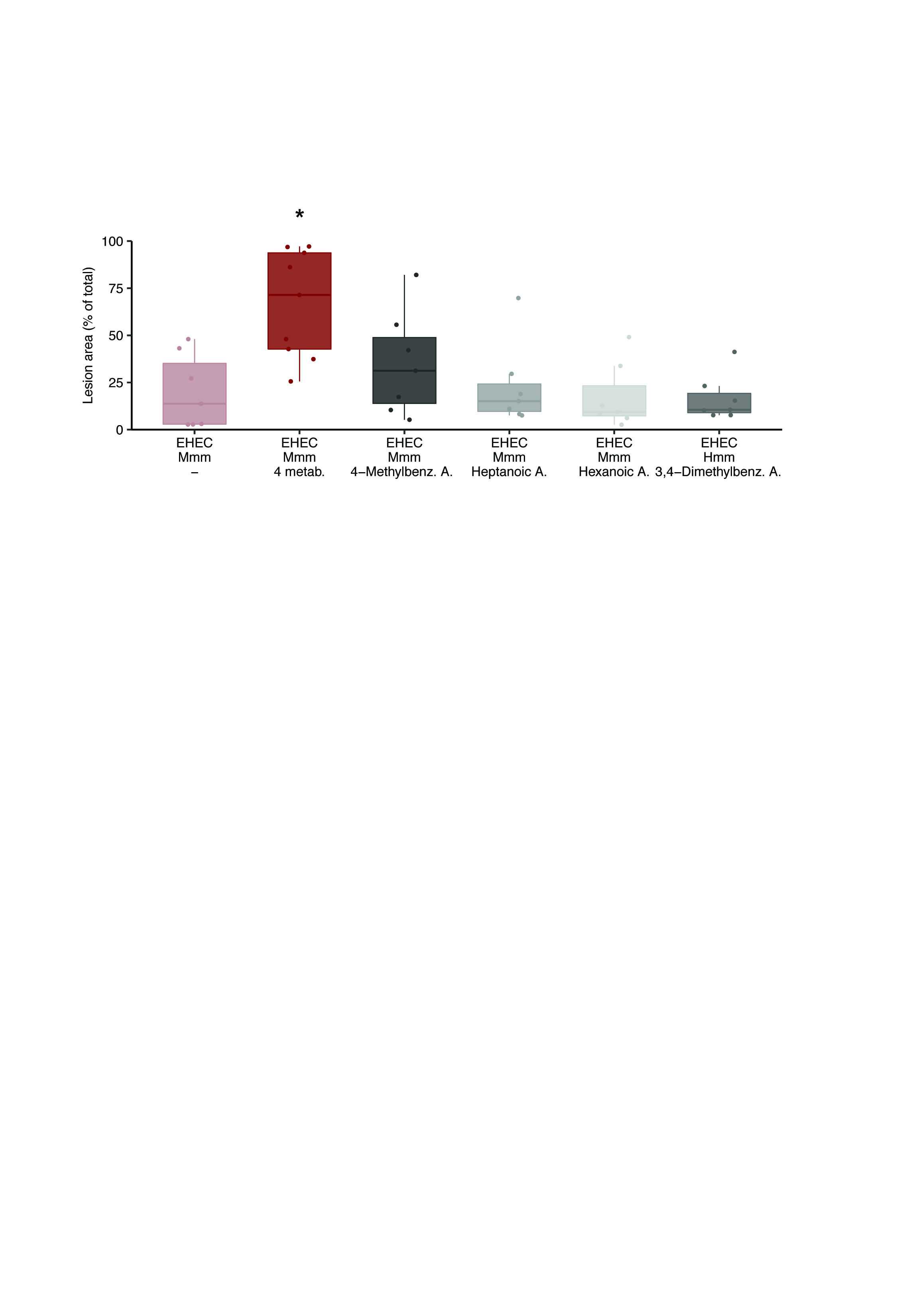

Supplement: Supplementary file 14 — Figure S12. The compound effect of the identified metabolites mediates increased pathogenicity. Effect of 3,4-dimethylbenzoic acid, 4-methylbenzoic acid, hexanoic acid, heptanoic acid individually and together (4 metab.) on epithelial injury in the Colon Chip with Mmm. Quantification of epithelial lesion area size. Data compounded from 3 experiments. *p < 0.05. (TIF 1541 kb) [file 40168_2019_650_MOESM14_ESM.tif]
